# Numerical Study on the Fatigue Limit of Metallic Glasses under Cyclic Tension-Compression Loading

**DOI:** 10.3390/ma13071732

**Published:** 2020-04-08

**Authors:** Jinfeng Yan, Wenjun Meng, Zhi Chen, Hong Guo, Xianguo Yan

**Affiliations:** 1 School of Mechanical Engineering, Taiyuan University of Science and Technology, Taiyuan 030024, China; mechenzhi@tyust.edu.cn (Z.C.); hongguo1@tyust.edu.cn (H.G.); yanxianguo@tyust.edu.cn (X.Y.); 2 China International Engineering Consulting Corporation, Ltd, Beijing 100048, China; 3 School of Mechanical Engineering, Shanxi Institute of Energy, Taiyuan 030600, China

**Keywords:** metallic glass, shear band, fatigue life, free-volume theory, hydrostatic stress

## Abstract

Numerical study was performed to determine the fatigue limit of metallic glass under tension-compression cyclic loading. A revised free-volume theory which considers the hydrostatic stress was utilized to make the predictions. Systematical simulations showed that a higher strain amplitude is prone to making the sample completely damaged earlier. However, lower strain fluctuations could result in a longer fatigue life. Shear banding evolution history described by free-volume localization could reasonably explain the mechanical responses of different samples. In addition, compressive loading could give rise to a higher stress than that under tensile loading because of hydrostatic stress contribution. In the end, a correlation between fatigue life and applied strain amplitude was plotted which could supply a guidance for designing the engineering application of metallic glass under periodic loading.

## 1. Introduction

Metallic glass is one kind of glassy family which possesses outstanding properties such as high strength and superior elastic limit [1,2]. However, it also exhibits very limited plasticity and poor fatigue properties [3,4] because its very thin shear band usually makes a sample fracture rapidly without a noticeable plasticity after yielding. In engineering, fatigue limit is an important parameter which could evaluate the sustainability of materials undergoing cyclic loading. Experimentally, Gilbert et al. [5] investigated the fatigue behavior of Zr-based metallic glasses by using four-point bending fatigue test, it was found that the fatigue endurance limit was about ~0.08 of ultimate tensile strength (UTS). Besides, Gludovatz et al. [6] found that the fatigue endurance limit for Pd-based metallic glass could be as high as ~0.48 UTS, which is very close to the values for most high-strength metals. Wang et al. [7,8,9] studied the fatigue behavior of Zr-based metallic glasses under high-cycle tension-tension loading. It was found that the fatigue limit is dependent on the micro-structures in metallic glasses, such as the porosity and conclusions. The low fatigue limit usually takes place when the volume of defect is high. In general, the fatigue endurance limit of most metallic glass is relatively low in a rather large range [5,7,8,9]. Therefore, its low fatigue property has put a big barrier in using it as an engineering material and it is necessary to understand its deformation mechanism under cycle loading. 

On the other hand, numerical method has been widely used to analyze deformation process and reveal the in-depth mechanism behind experiments. Since it is low-cost, several numerical investigations have focused on the fatigue behaviors of metallic glasses. By means of molecular dynamics (MD), Cameron [10] performed atomic simulations on metallic glass under cyclic fatigue loading. It indicated that the free-volume location is the key factor governing the shear band mechanism. In addition, Lo et al. [11] found that the Zr-based metallic glass could be very resistant to fatigue amplitude in small size scale after a systematical MD simulation for metallic glass under cyclic loading. Another work by MD simulation found that the localization of shear transformation zone obeys a power law with rate under tension-compression fatigue simulation [12]. By finite element method (FEM), Jiang [13] carried out a systematical simulation by classic free-volume theory to elucidate the damage mechanism in bulk metallic glasses. It was concluded that the fatigue endurance limit relied on different applied strain amplitude and cycling frequency. 

Although the above simulations have shed light on the shear deformation mechanism of metallic glasses under cyclic loading, it is not complete. Recently, it was found that hydrostatic stress played an important role in affecting the shear banding process which could lead to asymmetry of tension and compression [14,15,16,17]. It was argued that the hydrostatic stress should not be negligible, especially under multiple stress states. However, until now, few numerical investigations have been reported on the fatigue deformation process in metallic glass with the consideration of hydrostatic stress. Therefore, in this work, employed by the free-volume theory proposed by Zhao et al. [17], the deformation process as well as the free-volume evolution under tension-compression fatigue loadings were systematically investigated by FEM with the aid of ABAQUS software (ABAQUS 6.13-1, SIMULA Inc., Providence, RI, USA) and user-defined material subroutine (UMAT). The dependence of fatigue endurance limit on the different strain amplitude was illustrated and discussed. Furthermore, the detailed shear banding process was graphically described by free-volume localization. It is hoped that this work could provide an assistance in understanding the damage evolution for metallic glasses. 

## 2. Constitutive Laws 

Free volume theory [18,19] is a theoretical approach which has been accepted and recognized to quantitatively describe the shear band initiation and propagation in metallic glasses. The free volume aggregation is realized by a number of atomic jumps which could result in the onset localized shear bands. Mathematically, free volume is calculated by the departure from the ideally ordered structure and regarded as the difference between the average atomic volume in real material and that in the ideally ordered structure [18]. As discussed above, it was found that the compressive strength of metallic glass was higher than that under tension loading [16,17,20]. The underlying reason for this asymmetry could be addressed by the hydrostatic stress [17]. Tensile hydrostatic stress could assist in accelerating the localization of free volume, giving rise to relatively rapid formation of shear bands. In contrast, compressive hydrostatic stress could suppress the generation of shear bands by confining the movement of free volume. Therefore, in order to make a better prediction, the constitutive law used in reference [17] is utilized to simulate the damage process of metallic glass under tension-compression cyclic loading. From the micro-mechanism viewpoint, the net rate of free-volume increase could be displayed as [17]:

(1) $$\begin{array}{ll} \frac{\partial \bar{v_{f}}}{\partial t}= & \frac{\xi \Omega \left(1-2\upsilon \right)}{E}\frac{\partial \sigma_{kk}}{\partial t}+\\ & v^{*}f\exp\left[-\frac{\alpha v^{*}}{\bar{v_{f}}}\right]\exp\left[-\frac{\Delta G^{m}}{k_{B}T}\right]\left\{\frac{2\alpha k_{B}T}{\bar{v_{f}}C_{eff}}\left(\cos h(\frac{\tau \Omega }{2k_{B}T})-1\right)-\frac{1}{n_{D}}\right\} \end{array}$$

where f stands for the atomic vibration frequency, α is a geometrical factor of order 1, v^*^ is the critical volume (hard-sphere volume of an atom), $\bar{v_{f}}$ is the average free volume per atom, Ω is the atomic volume, E, υ are elastic modulus and Poisson’s ratio. $\Delta G^{m}$ is the activation energy, τ is the shear stress, $k_{B}$ is the Boltzmann constant and T is the absolute temperature, $C_{eff}$ expresses the effective elastic stress, typically it is expressed as $C_{eff}=E/3\left(1-\upsilon \right)$, $n_{D}$ is the number of atomic jumps needed to annihilate a free volume equal to v^*^ and is usually assumed as 3 [13,17,18,19]. According to the framework in reference [17], hydrostatic stress contribution could be quantitatively depicted by parameter ξ, which should be determined by comparing tensile and compressive strength obtained by FEM and experiments [13,17,21]. 

Furthermore, the total strain rate $\overset{\cdot }{\varepsilon_{ij}}$ could be decomposed as elastic part $\overset{\cdot }{\varepsilon_{ij}^{e}}$, plastic part $\overset{\cdot }{\varepsilon_{ij}^{p}}$ and hydrostatic stress induced strain rate $\overset{\cdot }{\varepsilon_{ij}^{h}}$ [17]:

(2) $$\overset{\cdot }{\varepsilon_{ij}}=\overset{\cdot }{\varepsilon_{ij}^{e}}+\overset{\cdot }{\varepsilon_{ij}^{p}}+\overset{\cdot }{\varepsilon_{ij}^{h}}$$

Including the hydrostatic stress separately in the constitutive model is a common mechanics and mathematics technique to deal with the condition when hydrostatic stress is noticeable enough to make its own distinct contribution [17].

The elastic part $\overset{\cdot }{\varepsilon_{ij}^{e}}$ could be inferred as [17]:

(3) $$\overset{\cdot }{\varepsilon_{ij}^{e}}=\frac{1+\upsilon }{E}\left(\overset{\cdot }{\sigma_{ij}}-\frac{\upsilon }{1+\upsilon }\overset{\cdot }{\sigma_{kk}}\delta_{ij}\right)$$

(4) $$\sigma_{e}=\sqrt{\frac{3}{2}S_{ij}S_{ij}},\;S_{ij}=\sigma_{ij}-\sigma_{kk}\delta_{ij}/3$$

In Equation (4), $S_{ij}$ is the deviatoric stress and $\sigma_{e}$ is the Mises equivalent stress. $\delta_{ij}=0\;\mathrm{when}\;i\neq j;\;\delta_{ij}=1\;\mathrm{when}\;i=j$. Besides, the flow equation $\overset{\cdot }{\varepsilon_{ij}^{p}}$ in Equation (2) as well as the free-volume evolution equation (Equation (1)) could be expressed as [17] by using the Mises stress criterion:

(5) $$\overset{\cdot }{\varepsilon_{ij}^{p}}=\exp\left(-\frac{1}{v_{f}}\right)\sin h\left(\frac{\sigma_{e}}{\sigma_{0}}\right)\frac{S_{ij}}{\sigma_{e}}$$

(6) $$\overset{\cdot }{v_{f}}=\frac{\xi \Omega \left(1-2\upsilon \right)}{\alpha v^{*}E}\overset{\cdot }{\sigma_{kk}}+\frac{1}{\alpha }\exp\left(-\frac{1}{v_{f}}\right)\left\{\frac{3\left(1-\upsilon \right)}{E}\left(\frac{\sigma_{0}}{\beta v_{f}}\right)\left[\cos h\left(\frac{\sigma_{e}}{\sigma_{0}}\right)-1\right]-\frac{1}{n_{D}}\right\}$$

in which, $\sigma_{0}=2k_{B}T/\Omega$ is the reference stress [17,19]; $\beta =v^{*}/\Omega$ and $v_{f}=\bar{v_{f}}/\alpha v^{*}$ is the normalized free volume [17,19]. 

Besides, the quantitative description for the hydrostatic stress contribution $\overset{\cdot }{\varepsilon_{ij}^{h}}$ is [17]:

(7) $$\overset{\cdot }{\varepsilon_{ij}^{h}}=\frac{\left(1-2\upsilon \right)\xi }{3E+\left(1-2\upsilon \right)\xi \sigma_{kk}}\overset{\cdot }{\sigma_{kk}}\delta_{ij}$$

## 3. FEM Simulations

The above constitutive laws in Equations (1)–(7) need to be implemented into ABAQUS software by means of UMAT to calculate the stress, strain and free volume distribution at every sub-step. The details on how these equations are implemented into ABAQUS could be traced according to the ABAQUS tutorial [22] and Zhao’s work [17]. In order to avoid the unnecessary computational cost without sabotaging simulation precision, a two-dimensional model [13,17,19,21] with fine enough meshes is employed here to analyze the fatigue response for specimen under cyclic loading. As in the work in references [13,17,19,21], the shear banding process is vividly described by the density and profile of the normalized free volume $v_{f}$. Because of the presence of hydrostatic stress, the plastic flow stress is a combined function of the effective Mises stress, the corresponding free volume and the hydrostatic stress. In the process of computation, all the variables such as stress and free-volume in each sub-step were automatically recorded in files for being utilized in further analyses and discussions.

**Figure 1:**
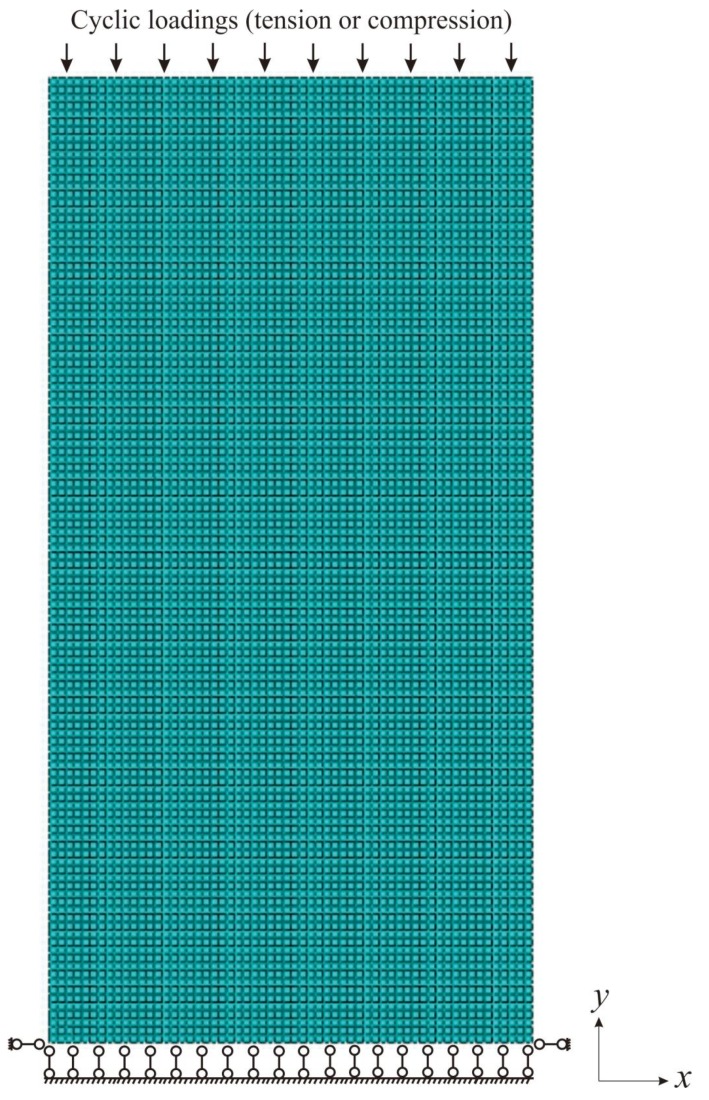
Illustration of the boundary conditions for sample used for fatigue simulations.

The sample used for fatigue simulation is 3 mm × 6 mm, as shown in Figure 1. Tension-compression cyclic loading is applied on the top end of specimen with the loading direction along *y* direction. In addition, Figure 1 shows the boundary condition in the FEM framework. Here, confinement is applied on the bottom line to prevent it from moving along the *y* direction. Besides, two ending points of the bottom line are fixed in the *x* direction. The well-meshed sample possesses 7200 elements and CPS4R (element type) is employed for all the samples. Besides, loading rate is fixed at 10^−5^ s^−1^. 

In the beginning of simulation, free volume field needs to be assumed. According to the free-volume theory, free volume distributes randomly within sample region [18]. It was assumed that the free volume distribution in the initial stage usually followed a Gaussian function [13,18,21]. Therefore, the free volume field could be illustrated according to the following equation [23]:

(8) $$v_{f0}\left(x,y\right)=v_{0}+\delta \exp\left(-\frac{{\left(x-x_{0}\right)}^{2}}{\Delta_{x}^{2}}-\frac{{\left(y-y_{0}\right)}^{2}}{\Delta_{y}^{2}}\right)$$

In Equation (4), the initial free volume $v_{0}$ is assumed to be 0.05; $\left(x_{0},y_{0}\right)$ is an arbitrary coordinate; δ is the amplitude of disturbance, and assumed as $v_{0}/10$ [23]; $\Delta_{x},\Delta_{y}$ are the characteristic half widths [23].

## 4. Results and Discussions

### 4.1. Parameters Determination

Before simulating the fatigue damage process of samples, it is necessary to determine the parameters used in FEM and make sure these coefficients are reasonable. Different from the Jiang’s work [13], hydrostatic stress contribution needs to be firstly considered by parameter ξ in equation (7). All the parameters in the constitutive model could be determined by comparing the stress-strain response in FEM and the previous experimental results until a promising precision is reached [21]. In more detail, the elastic modulus E and Poisson’s ratio υ are typically provided for individual material and $n_{D}$ is usually set as 3 [13,17,18,19]. Other parameters α, β, ξ need to be tuned in order to make a satisfying prediction on metallic glass under tension and compression. In this work, a Zr-based metallic glass [20] is used to demonstrate the numerical results. It was found that the tensile and compressive strengths of this material are 1.66 GPa and 1.84 GPa separately [20], displaying an obvious asymmetry of tension and compression. After computation, the material properties for this metallic glass are determined as: $E=96\mathrm{GPa},\;\upsilon =0.36,\;n_{D}=3,\;\alpha =0.75,\;\beta =1,\;\xi =0.1.$ is the specific parameter accounting for the hydrostatic stress contribution for this material. 

**Figure 2:**
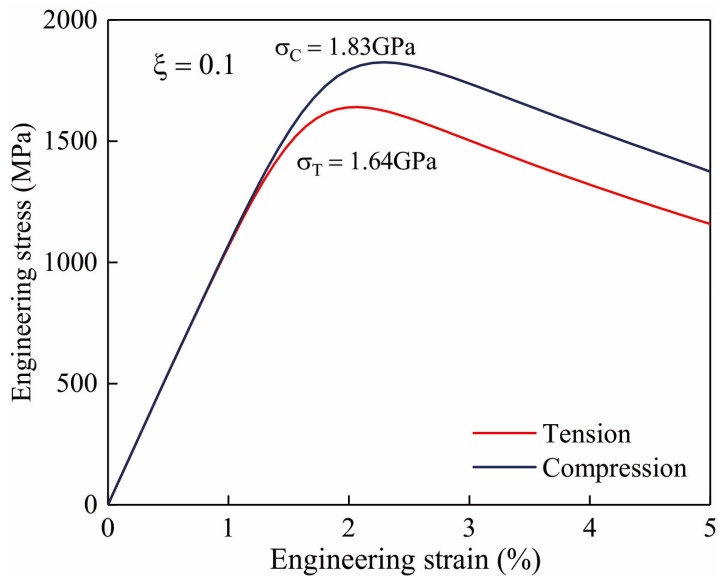
Simulated engineering stress-strain curves.

The simulated engineering stress-strain curves under uniaxial tension and compression are illustrated in Figure 2. Results show that the simulated yield strengths are 1.64 GPa and 1.83 GPa for tensile and compressive loading, separately. Here, engineering stress is calculated by the ratio of all the forces applied on the top end of sample (see Figure 1) to the top end area. Comparison with experimental results in reference [20] elucidates that the current parameters are good enough to be precisely describe the mechanical response for metallic glasses under uniaxial loadings. Because of the presence of compressive hydrostatic stress, the free volume is hard to be localized in terms of the suppression caused by multiple stress states, leading to a higher yield strength under compression. On the other hand, for both curves under tension and compression, there exists a declining part which follows the elastic and plastic stage. This descending part is the resultant effect of softening induced by free volume localization [18,19]. Doubtless, the softening process could also result in the damage behavior for metallic glass under cyclic loadings which will be discussed in detail below. 

### 4.2. Fatigue Spectra Used in This Work

**Figure 3:**
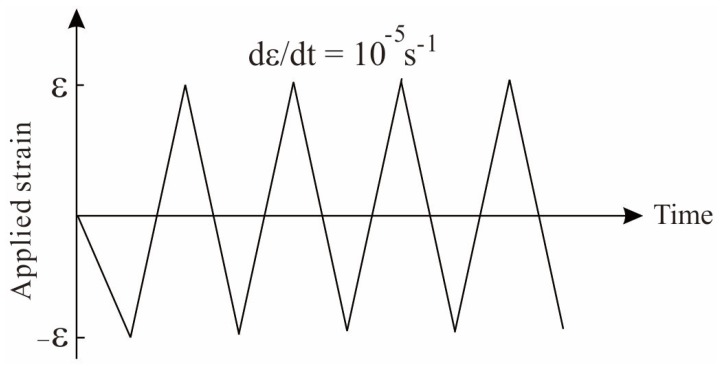
Tension-compression spectra used in fatigue simulations with different strain amplitudes. In this work, the cyclic strain amplitude ε is set to: 2.50%, 2.25%, 2.0%, 1.75%, 1.50%, 1.40%, 1.30%, 1.25%, 1.0%.

Displacement controlled scheme is used to exert fatigue loading on specimens. Figure 3 shows the fatigue loading patterns in which the engineering strain level ε are employed. In simulations, samples undergo cycle strains in the range from -ε to ε. The amplitude of strain ε is assumed as 2.50%, 2.25%, 2.0%, 1.75%, 1.50%, 1.40%, 1.30%, 1.25%, 1.0%. It covers the levels at which sample bears different elastic or plastic deformation processes. Therefore, it will be helpful to capture the strain amplitude effect on the damage process and fatigue life of metallic glasses. Besides, it should be mentioned that the positive strain denotes tensile loading and the negative one stands for the compressive loading. All the simulations are performed under the same strain rate 10^−5^ s^−1^ so as to investigate the fatigue process under low frequency condition. Since it will be time-consuming if these tests are all carried out by experiments, the related simulations could provide a fundamental guidance on its engineering design and application with very low cost.

### 4.3. Illustration of Deformation Process under Cyclic Loading

**Figure 4:**
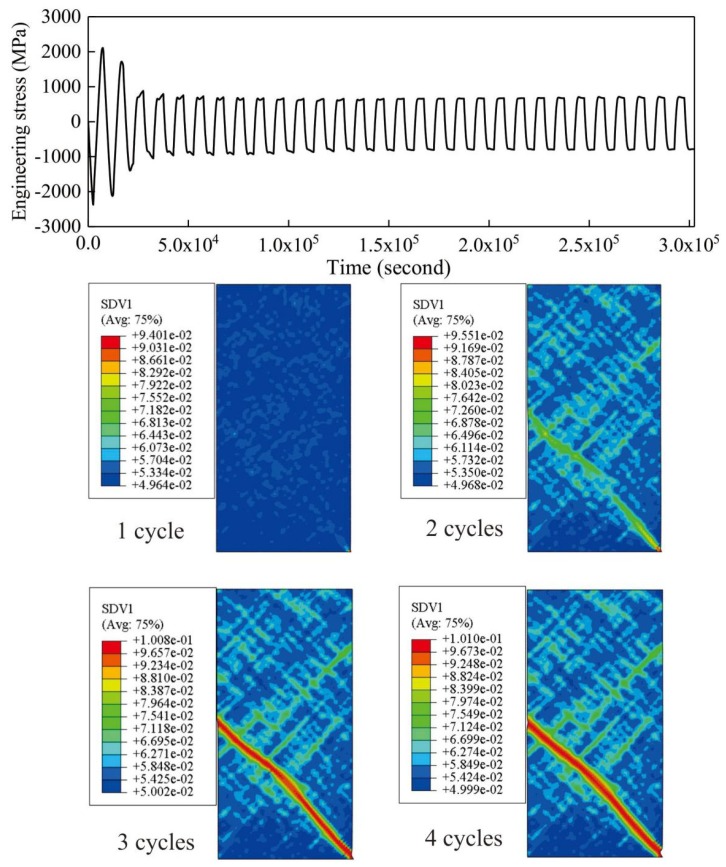
Damage evolution process with fatigue strain amplitude ε = 2.50%.

Actually, shear band is not a crack. There is an evolution process from shear band to crack. Since the crack is quite different from shear band, it could expected that a crack may affect the evolution of shear bands and the results could differ if the crack is separately incorporated in this model (for example, if surface separation is included in the model). Hereby, only shear band is modeled. In order to clearly understand the mechanical response and shear banding evolution under cyclic fatigue loading, Figure 4 shows the dependence of engineering stress on the total loading time, by taking the case under a strain amplitude ε 2.50% for instruction. It can be seen that the stress in the beginning is relatively high since there is no damage being caused. Along with the increasing time, stress fluctuates firstly with a descending amplitude. After about 3 cycles, the stress amplitude becomes stable and does not drop again. It could be understood that the declining amplitude in the first stage could be attributed to the cumulative damage in metallic glasses. That is: for longer times in this stage, stronger damage could be accumulated, leading to a lower bearing capacity. However, after certain cycles, since there is no damage being continuously accumulated, the stress becomes stable. Then the cycle number at which the stress becomes stabilized could be denoted as the fatigue limit according to reference [13]. Furthermore, careful observations could conclude that the stress under compression is higher than that under the corresponding tensile loading. This could be explained by the hydrostatic stress effect, which could be seen from the curve in Figure 2. Under compression, compressive hydrostatic stress could limit the formation of free-volume localization and bring up a relatively larger stress.

Then, the shear banding evolution after different cycles is illustrated to express the microscopic morphology during the damage process. After only one cycle, there are some small regions in which free-volume is localized; however, these localized regions have not been linked into one major shear band yet. When the sample has been subjected to two cycles, two large shear bands have been formed, and tend to coalesce together. Besides, there are plenty of small shear bands around the two major shear bands, suggesting that free-volume localization has spread out to most regions of sample which should be responsible for the loss of bearing capacity. After the sample has undergone three cycles, there is an obvious major shear band formed (red colored region) which runs through the entire specimen. It could be speculated that the specimen may probably fracture at this time. Furthermore, after four cycles, no big difference could be seen on shear banding morphology compared with the case after three cycles. This implies that most of the damage has been accumulated after three cycles and it could be regarded as the fatigue limit under this strain amplitude. It could be also verified by the stress-time curve in Figure 4. After three cycles, the stress amplitude is not able to drop noticeably. Instead, stress fluctuates quite stably within a certain range. This also proves the rationality of the definition of fatigue limits. 

### 4.4. Fatigue limits under Different Strain Amplitudes

#### 4.4.1. Effect of Applied Strain Amplitude

**Figure 5:**
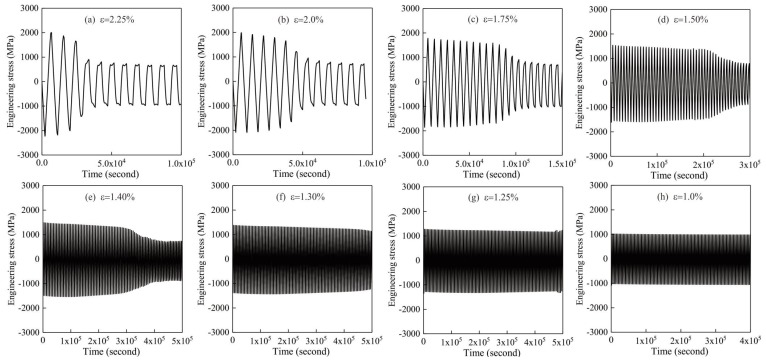
Correlation of engineering stress along with time under different strain amplitudes. (**a**) ε = 2.25%; (**b**) ε = 2.0%; (**c**) ε = 1.75%; (**d**) ε = 1.50%; (**e**) ε = 1.40%; (**f**) ε = 1.30%; (**g**) ε = 1.25%; (**h**) ε = 1.0%.

By using the method in Section 4.3, the fatigue limits under different strain amplitudes could be determined as long as the correlation of engineering stress and time is obtained. It should be pointed out that the ultimate fatigue limit in this work is assumed to 100 cycles according to investigation in reference [13]. Computation will be forcedly stopped if the sample is not able to be entirely damaged after 100 cycles in order to save computing time. Then, Figure 5 displays the relationship between engineering stress and the total time during fatigue process. As in the trend in Figure 4, stress amplitude firstly decreases, and then becomes steady when the fatigue limit is reached. With a larger strain amplitude, a lower fatigue limit could be found. However, for the samples with strain ε = 1.30%, 1.25% and 1.0%, there is no descending part found in curves, meaning that fatigue limit of these specimens is higher than 100 cycles. Besides, from all the curves, it could be realized that the compressive stress is higher than the tensile stress in the same loop, demonstrating a distinct asymmetry of tension and compression which is caused by the hydrostatic stress. This feature is different from the work in reference [13] in which no hydrostatic stress is considered. 

**Figure 6:**
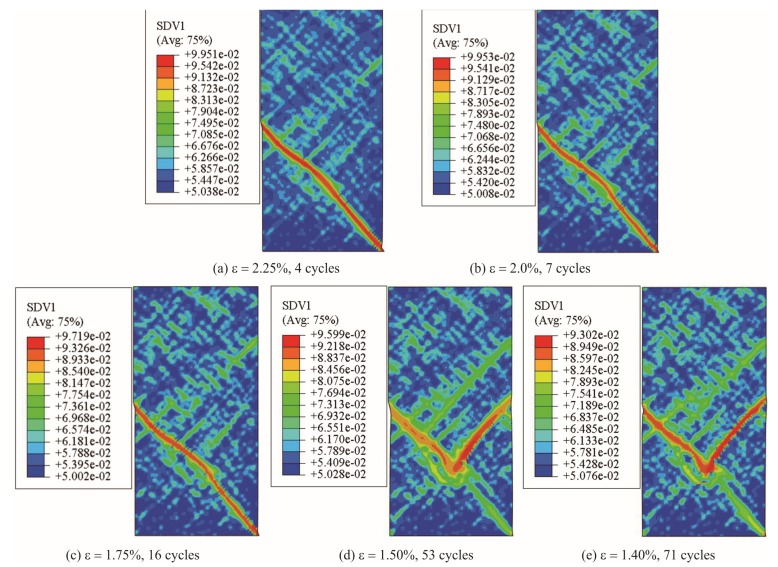
Contour plot of shear band morphology at fatigue limit. (**a**) ε = 2.25%, 4 cycles; (**b**) ε = 2.0%, 7 cycles; (**c**) ε = 1.75%, 16 cycles; (**d**) ε = 1.50%, 53 cycles; (**e**) ε = 1.40%, 71 cycles.

**Figure 7:**
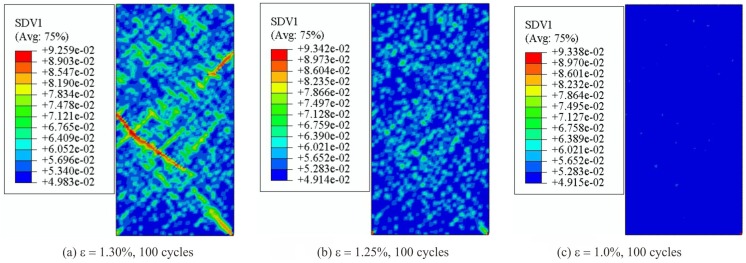
Shear band patterns for the samples after 100 cycles. (**a**) ε = 1.30%; (**b**) ε = 1.25%; (**c**) ε = 1.0%.

Furthermore, the contour plot of different samples at the time when fatigue limit is reached is shown in Figure 6. Since the fatigue limit for samples under strain amplitude ε = 1.30%, 1.25% and 1.0% is higher than 100 cycles, shear band morphology on these sample is excluded in Figure 6. It can be seen that mature shear bands have been formed within samples at the fatigue limit, implying an indication of complete damage of samples. Similar to the features in Figure 4, lots of small shear bands are found to distribute dispersedly around the major shear bands, suggesting a large area of softening behavior for these samples. Figure 7 shows the shear band patterns for the other three specimens (under strain amplitude ε = 1.30%, 1.25% and 1.0%) after 100 cycles. In Figure 7a, with a strain amplitude ε = 1.30%, it is shown that multiple shear bands have been developed in the sample. However, since no mature shear band is found in the specimen, there still exists extra room for the sample to continuously endure further fatigue cycles. According to its stress-time curve in Figure 5f, the stress only drops a small account and the sample has not been completely damaged yet. As seen in Figure 7b, with an even lower strain ε = 1.25%, fewer tiny shear bands are found so that there is no stress drop in its stress-time curve (in Figure 5g). When the strain amplitude ε = 1.0%, after 100 cycles, nothing is found in Figure 7c. This means that little damage has occurred on this sample even after 100 cycles. 

**Figure 8:**
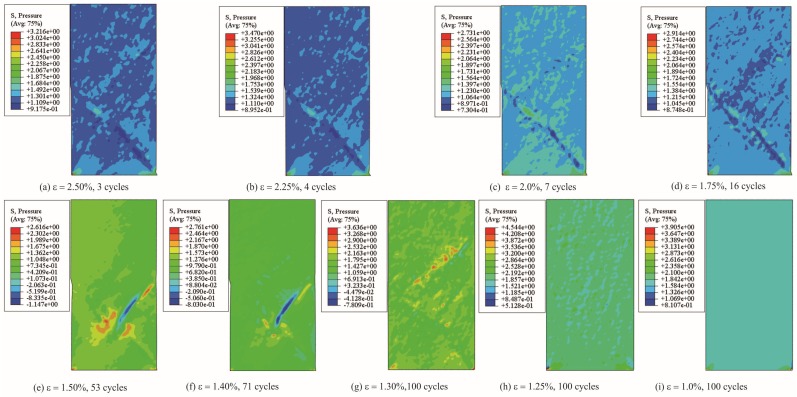
Hydrostatic stress distribution (described by “pressure” in ABAQUS) at fatigue limit or 100 cycles. (**a**) ε = 2.50%, 3 cycles; (**b**) ε = 2.25%, 4 cycles; (**c**) ε = 2.0%, 7 cycles; (**d**) ε = 1.75%, 16 cycles; (**e**) ε = 1.50%, 53 cycles; (**f**) ε = 1.40%, 71 cycles; (**g**) ε = 1.30%, 100 cycles; (**h**) ε = 1.25%, 100 cycles; (**i**) ε = 1.0%, 100 cycles. In which, the unit of pressure is actually denoted as $\sigma_{hydrostatic}/\sigma_{0}$ in which $\sigma_{0}$ is 225MPa (according to the deduction process in reference [19]).

As an important supplement, Figure 8 shows the hydrostatic stress distribution of samples at fatigue limit or 100 cycles. It should be mentioned that the unit of stress is actually denoted as $\sigma_{hydrostatic}/\sigma_{0}$ in which $\sigma_{0}$ is 225 MPa. The detailed deduction process could be traced from Reference [19]. It can be seen that the hydrostatic stress distribution is also dependent on the shear banding process. As seen in Figure 8i, hydrostatic stress distributes uniformly since very few shear bands initiate and the whole specimen is still at elastic deformation stage (see Figure 7c), even though it has undergone 100 cycles. However, at large strain levels in Figure 8a–d), the hydrostatic stress distribution is affected by shear band morphology (see Figure 4 and Figure 6a–c). In fact, compressive hydrostatic stress could partly impede shear band initiation by suppressing the formation of free-volume localization. However, this has limited influence on shear band once the shear band is formed.

In brief, it is shown that strain amplitude has a great impact on the fatigue limit. A larger range of strain fluctuations could yield short fatigue life. However, no damage will be generated even after a large number of cycles, for example, under the strain amplitude ε = 1.0%.

#### 4.4.2. Determination of Fatigue Limit

**Figure 9:**
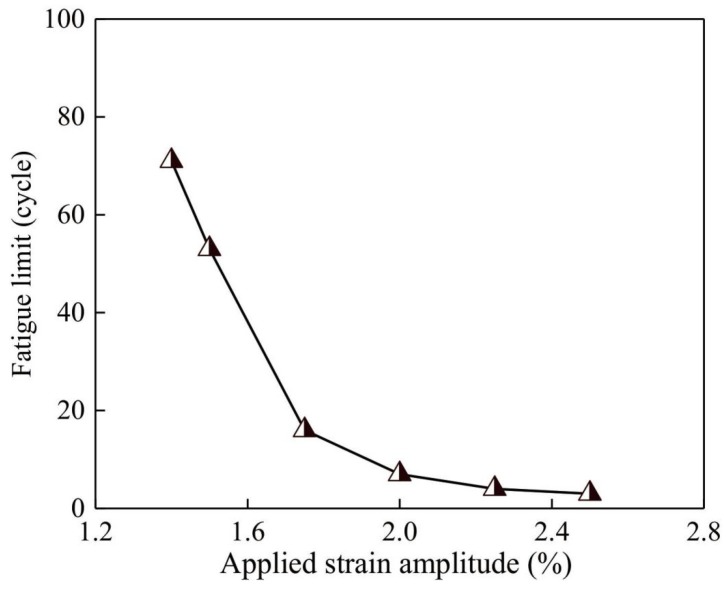
Fatigue limit as a function of applied strain amplitude. For the sample which has a fatigue limit higher than 100 cycles, data are excluded in this figure.

Based on the data obtained in Figure 4, Figure 5, Figure 6 and Figure 7, the correlation between fatigue limit and applied strain amplitude is plotted in Figure 9. For specimens with fatigue limit higher than 100 cycles, data are not included in this figure since computation will be stopped compulsively as illustrated above. It is clearly seen that the fatigue limit drops rapidly with the increased strain amplitude. When the strain amplitude is lower than 1.40%, a fatigue limit higher than 100 cycles should be expected. On the other hand, if the strain amplitude is higher than 2.5%, an even lower fatigue limit will be found. Furthermore, when the strain amplitude is large enough, the mechanical response ought to be identical to that under monotonic loading. Unfortunately, direction comparison between existing experiments and the current simulation could not be conducted because of the different applied loading modes. In the experimental results, the applied loading is lower than that in the current simulation so that the resultant fatigue limit is typically higher than 10^3^ cycles [7]. Besides, few previous experimental approaches are carried out by the tension-compression loading scheme. The purpose of this simulation is to provide a supplementary tool for understanding the fatigue mechanism of metallic glass with the consideration of hydrostatic stress. According to the simulated results, the quantitative trend is valid, which proves the availability of current work. In addition, this method may be useful for finding the endurance limit under high cycle fatigue. However, individual work must be performed before any conclusion is made. The obtained curve and relationship could provide a fundamental guidance when the metallic glass is used as a structure component which sustains periodic loading. 

## 5. Conclusions 

Fatigue limit for metallic glass under tension-compression cyclic loadings was determined by free-volume theory considering hydrostatic stress. Numerical simulations were performed by means of self-developed UMAT code in ABAQUS. Conclusions could be summarized as follows:

(1). Parameters being used in free-volume theory was ascertained and verified by previous experimental results. A satisfying agreement was achieved which could prove the rationality of the current model;
(2). Simulations on samples with different strain amplitudes were carried out to obtain the mechanical response and shear banding evolution. It was found that a higher strain amplitude could result in a lower fatigue limit, and vice versa. On the other hand, the shear band morphology could facilitate to explain the corresponding stress-strain response. Besides, compressive stress was found to be higher than the tensile stress in a same cyclic loop, displaying an obvious hydrostatic stress effect;
(3). Dependence of obtained fatigue limit on applied strain amplitude demonstrated that the fatigue limit decreased sharply along with increasing strain amplitude. This correlation could be quantitatively referred to when metallic glass is utilized as fatigue load parts.

## Nomenclature

$C_{eff}$	the effective elastic stress, typically we have $C_{eff}=E/3\left(1-\upsilon \right)$
*E*	Elastic modulus
f	the frequency of atomic vibration
t	time
T	the absolute temperature
α	a geometrical factor of order 1
$\overset{\cdot }{\varepsilon_{ij}}$	total strain rate
$\overset{\cdot }{\varepsilon_{ij}^{e}}$	elastic strain rate
$\overset{\cdot }{\varepsilon_{ij}^{p}}$	plastic strain rate
$\overset{\cdot }{\varepsilon_{ij}^{h}}$	hydrostatic stress induced strain rate
*v^*^*	the critical volume (hard-sphere volume of an atom)
ξ	Parameter accounting for hydrostatic stress contribution
$v_{0}$	Initial free volume
δ	the amplitude of disturbance
$\Delta_{x},\Delta_{y}$	the characteristic half widths
$\bar{v_{f}}$	the average free volume per atom
$v_{f}$	normalized free volume with $v_{f}=\bar{v_{f}}/\alpha v^{*}$
$\Delta G^{m}$	the activation energy
Ω	the atomic volume
$S_{ij}$	the deviatoric stress
$\sigma_{e}$	Mises equivalent stress
$\sigma_{kk}$	hydrostatic stress
$\sigma_{0}$	the reference stress
β	parameter defined as $v^{*}/\Omega$
υ	Poisson’s ratio
τ	normalized shear stress in microscopic model
$k_{B}$	the Boltzmann constant
$n_{D}$	the number of atomic jumps needed to annihilate a free volume equal to *v^*^*
